# *Yarrowia lipolytica* as a potential chassis for value-added products using a co-culture system and lignocellulose biomass-derived xylose

**DOI:** 10.3389/fmicb.2025.1704596

**Published:** 2026-01-12

**Authors:** Tahira Naz, Muhammad Tariq Saeed, Zujaja Umer, Waseem Safdar, Hassan Mohamed, Yuanda Song, Xiang Yu Zhao

**Affiliations:** 1 State Key Laboratory of Crop Biology, College of Life Sciences, Shandong Agricultural University, Taian, Shandong, China; 2 Colin Ratledge Center for Microbial Lipids, School of Agricultural Engineering and Food Science, Shandong University of Technology, Zibo, China; 3 Department of Diet and Nutritional Sciences, Ibadat International University, Islamabad, Pakistan; 4 Department of Biological Sciences, National University of Medical Sciences, Rawalpindi, Pakistan; 5 Department of Botany and Microbiology, Faculty of Science, Al-Azhar University, Assiut, Egypt; 6 School of Basic Medicine, Qilu Medical University, Zibo, China

**Keywords:** co-culture, genetic engineering, lignocellulosic biomass, MUFAs, xylose, *Yarrowia lipolytica*

## Abstract

*Yarrowia lipolytica,* a versatile and oleaginous yeast, has garnered significant attention as a promising microbial chassis for producing a vast array of important metabolic products, including trace minerals, vitamins, amino acids, protein and peptides, carbohydrates, and single-cell oil (SCO), primarily in the form of saturated high-value lipids like cocoa-butter equivalents and mono-unsaturated fatty acids (MUFAs). The US FDA has designated *Y. lipolytica* as a “safe-to-use organism” and given it GRAS (generally regarded as safe) classification for the synthesis of EPA, citric acid, and erythritol. Co-culturing multiple interspecies microorganisms together has proven to be a feasible and comparatively more efficient strategy than monoculture to target the degradation of waste components as a substrate and boost the production of significant metabolites. In recent years, a great deal of research has been devoted to exploring the potential of this host for the biosynthesis of valuable compounds from a wide variety of strategies. Despite ongoing efforts to improve our understanding of xylose metabolism in this yeast, there has been a notable lack of research focused specifically on the biosynthesis of natural products using xylose as a precursor, which is the second most abundant sugar in lignocellulosic biomass. This review also explores recent advances in the genetic modification of *Y. lipolytica* to enhance its ability to assimilate xylose and produce various secondary metabolites by using xylose as a substrate.

## 1 Introduction

One of the key determinants in establishing successful microbial processes at industrial scale is the careful selection of an appropriate production organism. *Yarrowia lipolytica* has attracted considerable attention as a promising chassis strain due to its high lipid accumulation potential and broad substrate utilization profile (Totlani et al., 2023). As a non-conventional yeast with a unique phylogenetic position and distinct genomic architecture, it offers several features desirable for biotechnology, including robust growth at high cell densities, stable and efficient genetic manipulation, strong stress tolerance, and overall process safety (Park and Ledesma-Amaro, 2023).

This versatile yeast occurs naturally in diverse ecological niches ranging from wastewater and soil to marine environments and fat-rich substrates and is renowned for producing a wide spectrum of metabolites. These include vitamins, amino acids, trace minerals, proteins and peptides, carbohydrates, and single-cell oils (SCO), particularly saturated and mono-unsaturated lipids such as cocoa-butter-equivalent fats and MUFAs (Bellou et al., 2016; Jach et al., 2020; Lopes et al., 2019). In addition, engineered strains have demonstrated remarkable capability in channeling metabolic flux toward acetyl-CoA, thereby enabling the synthesis of terpenes, lipid-derived molecules, and several metabolites originating from the pentose phosphate and shikimate pathways (Park and Ledesma-Amaro, 2023). Utilizing industrial, agricultural, and food wastes as feedstocks further enhances the economic appeal of *Y. lipolytica* while contributing to waste valorization and pollution mitigation (Dourou et al., 2018).

**Figure 1:**
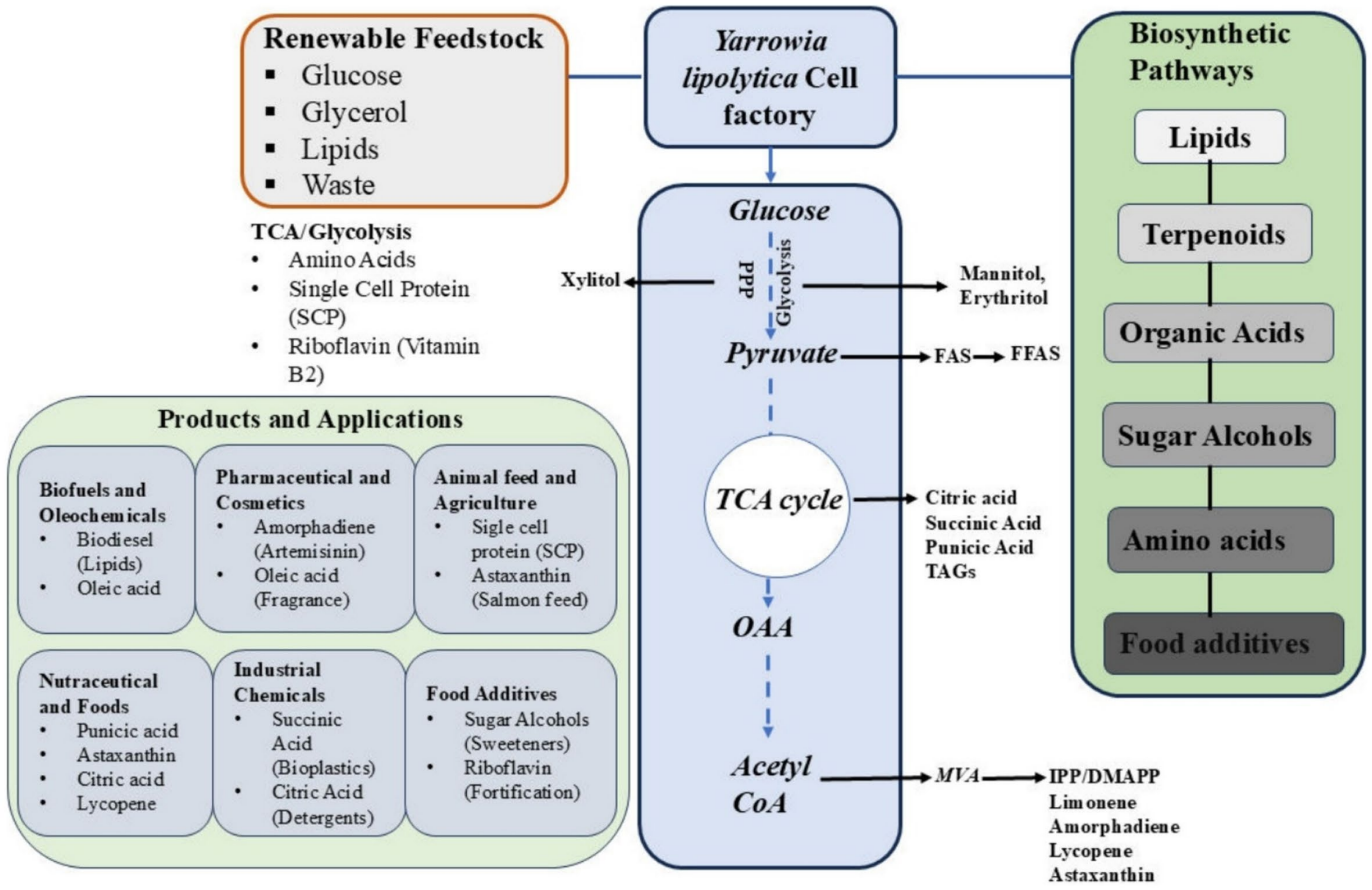
Industrially significant bioactive compounds and their derivatives produced by *Y. lipolytica.*

Safety is a critical consideration for any organism intended for industrial or food-related applications. The U.S. FDA has granted *Y. lipolytica* GRAS status for the production of EPA, citric acid, and erythritol, reinforcing its acceptability in commercial processes (Bilal et al., 2021). Beyond lipids, both wild-type and engineered strains have been developed to produce carotenoids, astaxanthin, limonene, sugar alcohols, vitamins, single-cell proteins, amino acids, and an array of bioactive compounds (Figure 1). Since its initial isolation, *Y. lipolytica* has also demonstrated utility in bioremediation of oil-contaminated water, soil, and various waste streams, simultaneously generating heterologous proteins and other valuable by-products (Gamraoui et al., 2023). In parallel, substantial progress has been made to expand its synthetic biology and metabolic engineering toolkit. *Y. lipolytica* harbors numerous homologous and heterologous DNA elements including promoters, terminators, localization tags, and selectable markers that facilitate efficient construction of expression cassettes and pathway engineering platforms. Advances in transformation techniques, including both chemical and electroporation-based methods, now support high-throughput screening and combinatorial engineering via library-based approaches (Larroude et al., 2018; Abdel-Mawgoud et al., 2018). Compared to other non-conventional yeasts, it therefore possesses a comparatively comprehensive and accessible engineering toolbox.

Despite its strengths, industrial biotechnology increasingly demands microbial platforms that are not only efficient but adaptable to complex substrates. Traditional monocultures often struggle with metabolic burden, limited substrate versatility, or insufficient product yields. Co-cultivation strategies where two or more microorganisms are intentionally paired offer a powerful solution to these limitations. Through functional division of labor, metabolic cross-feeding, and the sharing of physiological tasks, co-culture systems can enhance productivity, stability, and process efficiency while reducing the genetic load on individual strains (Li et al., 2024). *Y. lipolytica* stands out in these systems because it excels at converting intermediate and secondary metabolites into high-value fatty acids, terpenoids, polyols, and organic acids, even though it naturally lacks strong utilization of primary sugars such as xylose (Li et al., 2024).

This becomes particularly important when considering lignocellulosic biomass, where xylose represents the second most abundant sugar and a major determinant of process economics. Efficient xylose metabolism is essential for leveraging agro-industrial residues and biomass hydrolysates. While native xylose-utilizing organisms such as Scheffersomyces stipitis, *Escherichia coli*, and engineered *Saccharomyces cerevisiae* can convert xylose into central intermediates, pairing these microbes with *Y. lipolytica* in co-culture significantly broadens the range of potential value-added products (Gao et al., 2023). These include mono- and polyunsaturated fatty acids, sugar alcohols, carotenoids, and a variety of industrially relevant bioactive compounds. Thus, co-culture systems that combine efficient xylose utilizers with the metabolic strength of *Y. lipolytica* offer a promising and underexploited platform for lignocellulosic biomass valorization.

There are many reviews about strain improvement strategies to enhance the production of bioactive compounds in this yeast, however, several recent reviews published between 2022 and 2024 have briefly discussed *Y. lipolytica* within broader contexts of microbial consortia and mixed-culture bioprocessing, none have provided a dedicated, systematic, and comprehensive synthesis of the diverse co-culture strategies developed specifically for this organism. Existing reviews typically mention *Y. lipolytica* only as a secondary example, without detailing co-culture design principles, interaction mechanisms, metabolic division-of-labor, or application-specific optimization. Hence, this review summarized various co-culture systems of *Y. lipolytica* with other microbes. Despite ongoing efforts to improve our understanding of xylose metabolism in this yeast, there has been a notable lack of research focused specifically on the sugar in lignocellulosic biomass. This review also explores recent advances in the genetic biosynthesis of natural products using xylose as a precursor, which is the second most abundant secondary metabolites by using xylose as a substrate.

### 1.1 Co-culture: as a unique approach

Co-culture is defined as aerobic or anaerobic cultivation of various identified strains of microbes under sterilized environments. Principally, 99% of the microbes occurring in nature are in the form of consortiums, being involved in bioremediation and improvement of food quality and nutrition. However, artificially created co-cultures can fulfil more complex tasks by division of labour, which provides diverse intracellular conditions for various metabolic reactions (Diender et al., 2021). An important objective of growing two populations together is to combine specific species where an enzymatic activity occurring in one strain is absent from the other, and both species can grow mutually benefiting each other. This has emerged as a promising technique due to its effectiveness in industrial-scale processes (Ghosh et al., 2016). The health and well-being of a host are associated with its microbiota, not just in terms of composition but also in the interactions among different microorganisms (Kruger et al., 2019). They can be characterized as antagonistic, additive, or symbiotic interplays depending on the interactions occurring among the microorganisms from the same or different kingdoms. The survival rate and growth efficiency of one strain depend on the metabolites being produced by the other strain in the co-culture system (Ponomarova et al., 2017). Thus, the nutritional interactions among different members of a microbial community are the only factors that determine its stability and functionality.

In fermentation bioprocesses, monocultures have long been employed to generate a variety of bioproducts (Shahab et al., 2020). However, when it comes to using complex substrates such as lignocellulose biomass, using single strains may not be the most appropriate strategy, as the required characteristics are often not found in a single microorganism (Llamas et al., 2023). The exploitation of microbial consortia and co-cultures is becoming a focus of attention for many researchers to overcome technical barriers associated with single cultures. There is a long history of microorganisms being used with co-culture technology for the optimization of bioactive compounds and other components, adequate consumption of dense substrates and complex molecule biosynthesis (Hays et al., 2015). Integrated co-culture technology is a novel yet viable opportunity for the advanced engineering of biosynthetic pathways for improved production of desirable natural compounds. The inspiration for co-culture strategies came from several studies involving the innate interaction mechanisms among interspecies, increasing their survival rates and establishing adapted interaction systems between certain populations (Khan et al., 2018).

Among many innovative genetic engineering technologies for the increased bioproduction of diverse value-added bioactive compounds using monoculture, there are still many challenges to overcome. Monoculture fermentations face two significant challenges: navigating complex biosynthetic pathways and achieving efficacy, both of which require substantial metabolic engineering efforts and optimized cultivation conditions. The hurdles have been significantly prevented by using modular co-culture techniques (Wang et al., 2020; Zhang and Wang, 2016). Moreover, it has been reported in literature that biodegradation of waste material using a single micro-organism is challenging, which gives rise to the approach of co-culturing more than one microorganism together to increase the possibility of degradation of more complex food components. According to a study’s findings, the co-culture of *Y. lipolytica* can produce isoprenoid-related secondary metabolites more efficiently than a single culture when key enzymes are spatially localized and metabolic flux is adjusted in a modular fashion (Marsafari et al., 2022). Furthermore, researchers have discovered that by using co-culture strategies of different fungal species, the production of various degradative and oxidative enzymes involved in bioremediation can be significantly enhanced in comparison to monocultures (Yu et al., 2021).

### 1.2 Various strategies for co-culture system

**Figure 2:**
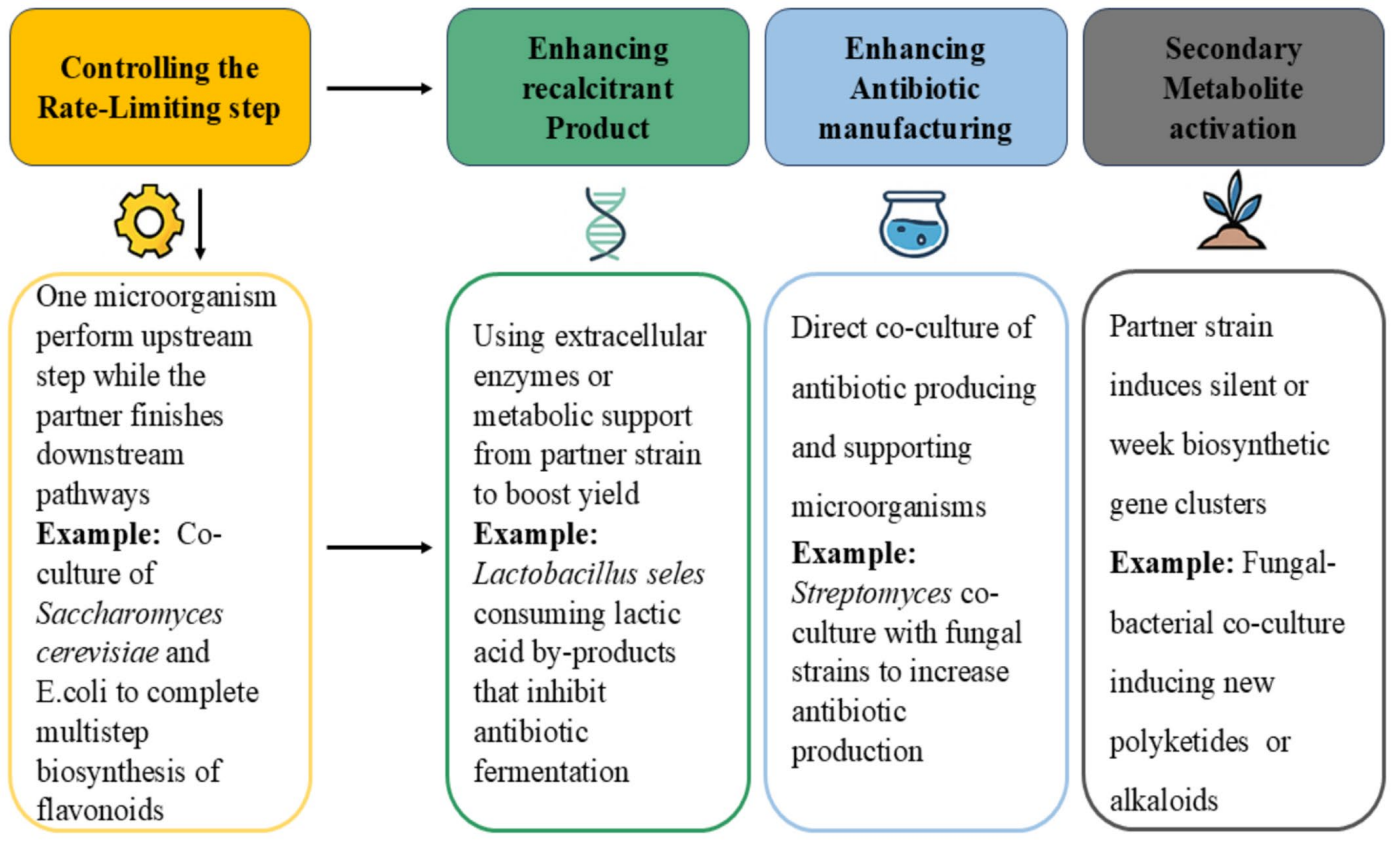
Co-culture strategies for enhancing microbial production of bioactive compounds.

In co-cultivation technology, microbes compete for nutrients and living space, producing a vast range of signals to communicate among them. These chemical signals are involved in culture growth as well as the production of secondary metabolites (Mao et al., 2020). Additionally, metabolic pathways in co-culture are also influenced by some particular enzymes and associated proteins (Kumar et al., 2019). Nowadays, scientists are focusing on developing strategies to increase lipid production and biomass yield with reduced production costs using fungal, algal, and bacterial consortia (Khan et al., 2018). In interspecies microbial co-cultures of two or more microorganisms, usually 6 different kinds of interactions take place: predation, competition, neutralism, cooperation, mentalism and commensalism. Positive interactions are always preferred over negative ones in a synthetic co-culture. Stable interactions are created when one microbe depends on the other for its members. Therefore, the features of a successfully formed microbial consortia include cell growth sustainability and performance of the required purposes efficiently and safely (Xu and Yu, 2021). Several co-culture strategies (Figure 2) have been utilized for improved yield and source utilization of *Y. lipolytica* with other microorganisms, such as; (a) controlling the rate-limiting stage involving one microbe handling the upstream and the other completing the downstream part of the biosynthetic process (Zhang and Stephanopoulos, 2016); (b) reducing the amount of biosynthetic enzymes being produced from by-products in which the second microorganism consumes the unwanted by-product that could lead to further complications (Chen et al., 2017); (c) using the extracellular enzymes of the supporting microorganism to improve the recalcitrant product for the primary microorganism (Asmamaw and Fassil, 2014); (d) improving or enhancing antibiotic synthesis by co-culturing of manufacturer strain and trial organisms (Sung, 2016). Moreover, cross-feeding or artificial quorum sensing (QS) circuit systems have designs managed to motivate mutual cohabitation, leading to a successful coexistence. The material-mediated approach presents opportunities for additional uses of synthetic microbial consortia in stable and effective chemical production and is a workable means of dividing up various niches (Jiang et al., 2023).

Studies on the interaction mechanisms of *Y. lipolytica* with several other species have been conducted, producing positive outcomes and proficient source utilization. For instance, to enhance the biotransformation of okra and pork lard, a co-culture between yeast and bacteria, *Y. lipolytica* and *Lactobacillus paracasei,* respectively, was employed as a technological tool. Metabolic engineering in modular co-culture systems acts by combining the strains that carry each pathway module in the engineered strains and creates a synthetic complex that can support various modules expressing functional genes in hosts to generate drop-in bio-products (Jawed et al., 2019).

Multiple approaches have been employed in co-culture systems such as; (a) to overcome undesired enzymes secreted as by-products during biosynthesis of a valuable product which were consumed by the second microorganism for their metabolism, (b) to support growth of primary organism through secreting antibiotics by the co-organism to eradicate the unwanted, (c) to supply substrate or enzymes for producer organism for biosynthesis of valuable product, (d) to carry out limiting steps during biosynthesis pathway for the producer organism by the second organism, (e) to support main microorganism in removal of inhibiting metabolites by the second microorganism during fermentation (Jawed et al., 2019). In the following section, co-cultivation of *Y. lipolytica* with different microorganisms and secondary metabolites production due to their symbiotic relationship in co-culture systems are described in detail and summarized in Table 1.

**Table 1:** Summary of the biosynthesis of valuable biochemicals produced and strategies involved by the co-culture system of *Y. lipolytica* with other microorganisms.

Type of co-culture	Microorganisms’ participation	Metabolite produced/function/	Increase yield/significant results	Growth effects	Strategy	References
Yarrowia- bacterium co-culture	*Y. lipolytica* and *L. lactis* sub sp. lactis	Nisin	50%	Growth of *L. lactis* increased by 49% with 4- fold increase	Utilization of lactate as a carbon source instead of sucrose	Ariana and Hamedi (2017)
	*Y. lipolytica and B. amyloliquefaciens*	Lipopeptides and fatty acids	470.24 mg/L	3.23-fold more growth as compared to pure culture	Recycling and bioconversion of food waste into value-added products by artificial microbial consortium	Bai et al. (2023)
			18.11 g/L			
	*Y. lipolytica, S. xylosus, and L. lactis*	Reduced amino acids and increased production of lactate	30-fold production of lactate	Growth of *Y. lipolytica* reduced in the presence of *S. xylosu*	Down-regulation of amino acid metabolism and over-expression of lactate dehydrogenase gene	Mansour et al. (2009)
	*Yeast (including Y. lipolytica) and Bacteria*	Aroma compound production	Production of 24 volatile aromatic compounds	–––––	Construction of 39 Co-culture of 3 yeasts, 3 *G. candidum,* and 5 bacteria	Martin et al. (2003)
	*Y. lipolytica and L. paracasei*	Biotransformation of Pork lard and Okara	Antioxidant potential 6.77–17.78 mM TE/g	–––––	Solid state fermentation	Cotârleț et al. (2020)
			Antimicrobial activity against *Aspergillus niger*			
Yarrowia- microalgae co-culture	*Y. lipolytica and Chlorella vulgaris (1:3)*	Lipid production	Lipid Content (w/w) 8%, Lipid (g/L) 0.12 Lipid productivity (mg/L/day) 60	1.54 g/L (CDW)	Liquid digestate of dairy wastewater and glycerol utilization	Qin et al. (2018)
	*Y. lipolytica and C. vulgaris (1:1)*	Lipid production	Lipid Content 0.31 g/L productivity 51.66 mg/L/day	1.62 g/L (CDW)	Construction of mixed culture for the treatment of liquid digestate of yeast industry	Qin et al. (2019)
	*Y. lipolytica and C. pyrenoidosa*	Biomass production	Carbohydrates 1.82 g/L Protein 1.99 g/L, Lipid 0.77 g/L	Biomass and growth increased	Mono and co-culture construction for faster cell propagation of microalga and yeast	Qin et al. (2019)
	*Y. lipolytica and C. pyrenoidosa*	Nitrogen removal from Wastewater	Elimination of biological nitrogen	–––––	Expression of cysteine dioxygenase, hypothetical protein, and histone-lysine, SETD1	Zhong et al. (2023)
					Up-regulation of ATP-binding cassette, (CFTR/MRP), member 10 (ABCC10)	
	*Y. lipolytica and C. vulgaris*	NH3-N and SO4 2 − removal from yeast industry liquid digestate	Biomass:1.39–1.56 g/L (5 times dilution)	Biomass enhanced significantly	Liquid digestate of dairy wastewater and glycerol utilization	Qin et al. (2019)
			1.23–1.53 g/L (10 times dilution)			
			Lipid yield: 0.073–0.154 g/L (5 times dilution)			
			0.112–0.183 g/L (10 times dilution)			
	*Y. lipolytica and C. pyrenoidosa*	Anti-hemolytic activity in oxidative stress	Anti-oxidative associated amino acids in the protein	–––––	Extraction of protein	Wang et al. (2019b)
		Trypsin-hydrolyzed peptides	Oxidative stress in HepG2 cells		Study amino acid structure by (QSARs)	Liu et al. (2019a)
Yarrowia- yeast co-culture	*Y. lipolytica and S. cerevisiae*	Fatty acid ethyl esters	FAEEs 4.8 mg/L, Ethanol 70.8 mg/L	–––––	Co-overexpression of PDC1, ADH1, wax ester synthase (MhWS)	Yu et al. (2020)
	*Y. lipolytica and C. rugosa*	Biodegradation of palm oil mill wastewater	Removal of phenolic content 36%, Triglyceride 98.5%, COD 60.3%	–––––	Crude extracellular enzyme preparations	Theerachat et al. (2017)
	*Y. lipolytica (Po1f and Po1g strains)*	Amorphadiene	71.74 mg/L	Endoplasmic reticulum surface area enhanced	Stimulate the ADS gene and enlargement of ER surface area	Marsafari et al. (2022)
	*Y. lipolytica and T. reesei*	Erythritol	267.1 mg/gds	–––––	One pot solid state fermentation of distillers grains (DGS)	Liu et al. (2022)
	*Y. lipolytica and T. cutaneum*	Bio-reduction of tellurite and selenite	Removal of Te and Se containing Te–Se NPs Size: 25 to 171 nm	–––––	Production of Te–Se NPs	Hosseini et al. (2023)
	*Y. lipolytica and Trichoderma reesei*	Conversion of Cellulose into Citric Acid	Citric acid 83.4 g/l Isocitric acid 8.7 g/L Under specific condition	–––––	Pre-treated straw cellulose direct converted into Citric Acid	Liu et al. (2014)

### 1.3 Co-culture system of *Y. Lipolytica* with bacteria

*Y. lipolytica,* known for its exceptional lipid accumulation capabilities, can efficiently utilize diverse carbon sources, while bacteria contribute through their metabolic prowess, offering an advantageous mix of enzymatic activities. Co-culturing multiple interspecies microorganisms has proven to be a feasible and comparatively more efficient strategy than monoculture to target the degradation of waste components as a substrate and boost the production of significant metabolites (Bai et al., 2023). In a culture system involving both bacterial and fungal strains, the function of the fungus varies. The fungus can act as a host strain in some circumstances, and in other conditions, its actions may differ. The particular mutualistic or antagonistic association between the two organisms determines the specific function performed by the fungal strain (Lastovetsky et al., 2020). There is a massive variety of secondary metabolites and valuable products being sourced from fungal–bacterial co-culture utilized by several industries as natural raw materials. A study focused on the generation of aroma/odor compounds in cheese using co-culture among various yeast strains (including *Y. lipolytica*) and bacteria exploited in almost 39 different cultures. Results revealed a significant difference in the production of 24 volatile substances (alcohols, aldehydes, esters, sulfides, terpenes) compared with pure cultures (Hudecová et al., 2009). Mansour et al. (2009) investigated the association of *Y. lipolytica, Staphylococcus xylosus,* and *Lactococcus lactis* as a co-culture system and reported their interaction. They studied yeast-bacterium interactions using artificially designed medium and different cultures focusing on glucose, lactate and amino acid regulating genes. The production of lactate in a co-culture of three microorganisms increased lactate levels by 30 times compared to a pure culture of *Y. lipolytica* due to the influence of the bacterial expression system on the expression of the dehydrogenase gene in *Y. lipolytica*. Another study utilizing artificial microbial consortium (AMC) between *B. amyloliquefaciens* HM618 and recombinant *Y. lipolytica* YL21 focused on the synthesis of higher-level fatty acids to bio-transform food waste into lipopeptide and fatty acid co-productions. Findings showed promising results and significant improvement in lipopeptide production of the exogenous fatty acids along with an increase in lipopeptides, i.e., fengycin, surfactin, and iturin up to 7.24, 12.13, and 3.23 times, respectively, as compared to pure culture of *B. amyloliquefaciens* (Bai et al., 2023). *L. lactis* is a potential producer of nisin, a bacteriocin being utilized commercially in many high-end industries. Lactate accumulation in fermentation media inhibits the growth of *Lactobacillus lactis,* consequently lowering the yield of Nisin. This obstacle was effectively addressed by cultivating the organism alongside *Y. lipolytica*, which promoted increased nisin production by metabolizing lactate. In a co-culture system using molasses based medium *Y. lipolytica* has been reported to consume not just sucrose as a sole carbon source but lactate as well reducing the lactic acid titer up to 10% in the media, boosting nisin output and growth of *L. lactis* up to 50 and 49%, respectively, as a compared to the pure culture (Ariana and Hamedi, 2017).

Another application of *Y. lipolytica* and other bacterial species forming a synthetic consortium is their utilization as a biotechnological tool for the biotransformation of various food products, allowing for the evaluation of the products’ post-fermentation antioxidant and antibacterial properties. The study reported by Cotârleț et al. (2020) used *Y. lipolytica* and *L. paracasei* in a co-culture system via solid-state fermentation to biotransform pork lard and Okara. Two *Y. lipolytica* strains were constructed for co-cultivation with *L. paracasei* or for independent fermentation. In order to institute the special effects of fermentation factors that are acceptable to obtain a fermented item for consumption, modified antioxidant and antibacterial activities were applied using the Plackett–Burman tentative technique. The findings demonstrated that fermented products had an antioxidant potential of 6.77–17.78 mM TE/g and 24–64% antibacterial activity against *Aspergillus niger.* It was concluded by the findings that solid-state fermentation in a consortium enhanced the functional properties of pork lard and okra, providing us with an innovative and practical solution for their disposal while producing value-added fermented products (Cotârleț et al., 2020).

Furthermore, researchers have successfully introduced a developed microfluidic-biosensor co-culture system. In a recent study, a technique for co-culturing *E. coli* EryD-bio-sensing and erythritol-producing *Y. lipolytica* cells in pico droplets was developed, which enabled high-throughput screening of erythritol mutations with high yields (about 10^5^ cells/h). In a 5 L bioreactor, encapsulating yeast cells with erythromycin resulted in a significant increase in erythritol yield and production rates of 16.97 and 26.09%, respectively (Li C. et al., 2023; Li S. et al., 2023).

### 1.4 Co-culture system of *Y. Lipolytica* with microalgae

Microalgae have been used as a feed additive worldwide for many years since microalgal biomass contains a significant number of polysaccharides, protein, unsaturated fatty acids, cytochromes and other valuable bioactive compounds that are extensively utilized in animal feed. It has been reported that the saturated fat contents of meat, eggs, and milk can be significantly improved by feeding the animals with yeast or algal biomass (Liu et al., 2019b). Yeast-algae is a promising animal feed additive due to its potential benefits; however, its application is currently restricted by its low yield and high cost. The cost of culture technology has significantly reduced in recent years, particularly through the research of the yeast-microalgae co-culture system. Researchers have been focusing on co-culturing algae and yeast to improve microalgal biomass economically (Zhong et al., 2023). Microalgae have been observed to facilitate the metabolic processes of yeast by generating oxygen through photosynthesis. In return, yeast breaks down complex macromolecules of organic substrates into smaller molecules and CO_z_, thereby improving the growth of algae (González et al., 2008). The symbiotic association between *Y. lipolytica* and algae in a co-culture system has been reported for various biotechnological applications. For example, the co-culture of *Y.lipolytica* and *Chlorella* or *Scenedesmus* can potentially remove nutrients effectively from any growth medium. This co-culture approach is especially useful in wastewater treatment systems because the algae assimilate nutrients like phosphorus and nitrogen while the yeast can effectively absorb organic carbon sources (Qin et al., 2019). For example, liquid digestate treatment in conjunction with microalgae biomass production is reported to be the best way to utilize liquid digestate resources (Xia and Murphy, 2016).

A co-culture comprising *C. vulgaris and Y. lipolytica* in a 1:3 ratio was established using liquid digestate of dairy wastewater (LDDW) as a growth medium and glycerol as the carbon source. The comparative analysis between monoculture and mixed culture revealed that the co-culture demonstrated enhanced nitrogen and phosphorus consumption, resulting in increased yields of biomass (1.62 g/L), lipid (0.31 g/L), and protein (0.51 g/L) compared to the mono-culture of *C. vulgaris* (Qin et al., 2018). Transcriptional analysis of genes involved in nitrogen assimilation revealed that transcript levels of nitrate reductase and glutamine synthetase II genes in *C. vulgaris* were reduced in co-culture as compared to monoculture in the presence of sufficient ammonium.

The combined co-culture approach achieved notably faster microalgae and yeast cell growth, smaller individual yeast cell size and higher microalgae chlorophyll levels. The co-cultured cells facilitated carbon and nitrogen absorption and directed carbon flux towards carbohydrates. In addition to a higher yield of lipid (0.77 g/L), carbohydrates (1.82 g/L), protein (1.99 g/L) and energy value (114.64 kJ/L), the harvested mixed microbial biomass has greater potential applications in renewable energy, nutrition, and industrial use (Qin et al., 2019). Co-cultures of *Y. lipolytica* and algae have also been explored for biofuel production, exploiting the oleaginous nature of the yeast and the fast growth rate of algae. The rapid generation of biomass poses a notable challenge in microbial lipid production. This concern was addressed by establishing a co-culture of *Y. lipolytica and C. pyrenoidosa*. Their study aimed to compare the biomass production between the pure cultures of *Y. lipolytica* and *C. pyrenoidosa* and a mixed culture involving both microorganisms (Qin et al., 2019). Qin et al. (2019) also investigated this co-culture combination to treat liquid digestate from the yeast industry, along with the co-production of biofuel. This study also focused on specifications of cell growth, nutrient removal efficiency, and energy storage capacity in both mono and co-culture systems. They found that the co-culture showed increased lipid production and a higher heating value compared to the monoculture. Furthermore, the co-culture system demonstrated elevated removal rates for NH_3_-N and SO4^−2^ compared to the pure culture.

More recently, Zhong et al. (2023) established a co-culture system of *C. pyrenoidosa* and *Y. lipolytica* in different ratios for 6 days to assess the potential for nitrogen removal from wastewater and understand and optimize the associated metabolic processes. They found that the co-culture system in a 3:1 ratio was optimal in removing Total N_2_, COD and NH_3_-N in 2–6 days with decreased nitrogen content as compared to the control group. Analysis of mRNA and microRNA (miRNA) expression in the co-culture of *C. pyrenoidosa* and *Y. lipolytica* at 3 and 5 days revealed 9,885 and 3,976 differentially expressed genes (DEGs) for the respective time points. The co-culture system of *C. pyrenoidosa* showed upregulation of many genes associated with carbon metabolism, nitrogen metabolism, amino acid synthesis, and various transporter proteins. It was assumed that *Y. lipolytica* in coculture may act as a stress inducer to stimulate carbon and nitrogen removal, amino acid assimilation, and glucose metabolism, further activating the miRNAs cascade. The differentially expressed profiles of miRNA/mRNA confirmed the synergistic effects of a coculture system on waste disposal.

This kind of co-culture system was also developed for some protein production with higher antioxidant activities. For instance, Wang et al. (2019a) showed the competitive relationship between *C. pyrenoidosa* and yeast in a coculture system, with the algae exerting dominance and eventually rendering the yeast undetectable in the mixed system. Nevertheless, it was noteworthy that during the co-culture period, the yeast exhibited a substantial increase in protein content compared to a monoculture of *C. pyrenoidosa.* The extracted proteins from coculture in different ratios provided insight into structure-related detoxifying effects against oxidative stress induced by 2,2′-azobis (2-methyl-propanimidamidine) dihydrochloride. It was observed that these ratios did not have an impact on the stability of dissolved oxygen but significantly increased the yield of chlorophyll A and total protein within 5 days of co-cultivation. The co-cultivation of these microorganisms resulted in the production of protein hydrolysates with high antioxidative activity. This enhanced antioxidant potential was attributed to a specific structure of protein extracted from the co-culture, which showed exposed hydrophobic residues on the surface. Additionally, the protein from co-culture exhibited potent anti-haemolytic activity under oxidative stress as compared to the mono culture of *C. pyrenoidosa*. In the same way, Liu et al. (2019a) characterize the amino acid sequence of trypsin-hydrolyzed peptides (EHPs) by quantitative structure–activity relationships (QSARs) model extracted from a symbiotic coculture system of *C. pyrenoidosa* and *Y. lipolytica.* The research showed that EHPs from co-culture demonstrated strong protective effects against oxidative stress in HepG2 cells, potentially due to their structures. However, the mechanism by which these peptides exert their antioxidant activity is still unknown.

### 1.5 Co-culture system of *Y. Lipolytica* with other yeasts

*Y. lipolytica* is characterized by its ability to produce bioactive compounds on an industrial scale when co-cultivated with other yeasts. This is attributed to its versatility in utilizing different carbon sources such as glucose, fructose, mannose and galactose. In addition, *Y. lipolytica* exhibits high tolerance to elevated substrate concentrations and increased productivity and is more amenable to genetic modification using molecular techniques. Co-culture with different types of *Yarrowia* can also biotransform the waste resources into valuable products. Citric acid is a multipurpose industrial acid that is widely used in preservation, as a buffer, emulsification, and antioxidant in food, cosmetics, and pharmaceutical industries. *Aspergillus niger* was the first organism being used for the production of citric acid, while *Y. lipolytica* has more ability to produce citric acid due to diverse substrate utilization and feasible modification. Previous studies have proven that massive carbon sources like fruit extras, wastes from industries, and inulin (Liu et al., 2010), can produce citric acid by providing a limited nutrients environment (Chi et al., 2011). Liu et al. (2014) constructed a mixed culture of free cells of *Y. lipolytica SWJ-1b* and immobilized mycelium of *Trichoderma reesei* for the production of citric acid by using raw straw (inexpensive and renewable) cellulose. *T. reesei* can produce cellulase and make it possible for *Y. lipolytica* to convert cellulose into citric acid. Simultaneous saccharification and fermentation (SSF) were used to avoid the inhibition of cellulase yield, resulting in 10.7 g/L of citric acid production from 40.0 g/L straw and 32.8 g/L of citric acid from 20.0 g/L glucose. However, co-culture showed 83.4 g/L and isocitric acid (8.7 g/L) from 100.0 g/L straw and the addition of glucose 50.0 g/L.

Amorphadiene is a forerunner for the production of artemisinin, which is an antimalarial drug that is obtained from the plant (*Artemisia annua*) extraction, produced in smaller amounts and is expensive (Martin et al., 2003). Microbial metabolic engineering and synthetic biology methodology offer an alternative to plants for the synthesis of amorphadiene. Microbial mix culture can provide a platform to attain amorphadiene production cost-effectively. Amorphadiene production was significantly improved in recent research by co-culturing two different strains of *Y. lipolytica* (Po1f and Po1g) through genetic modifications. In addition to co-cultivation, subcellular localization of the ADS gene (amorphadiene synthase) to the endoplasmic reticulum, simultaneous utilization of mixed carbon sources, and expansion of the surface area of the endoplasmic reticulum resulted in 71.74 mg/L of amorphadiene production. The findings indicate that a co-culture system involving *Y. lipolytica* demonstrates greater efficiency compared to a mono-culture in the modular biosynthesis of isoprenoid-associated secondary metabolites (Marsafari et al., 2022),

Recently, *Y. lipolytica* was considered as a competent organism for the production of erythritol (Paulino et al., 2021) due to its ability to utilize different carbon sources and raw materials as an inexpensive substrate (Rakicka et al., 2017). Erythritol is a calorie-free sweetener which abundantly occurs in fruits, seaweeds, fungi and also in fermented foods. The human body has acceptance of erythritol, and it is easily absorbed and eliminated and prevents the increase of insulin (Hijosa-Valsero et al., 2021). A co-culture system between *Y. lipolytica* and *T. reesei* was also constructed for the production of erythritol by using distiller’s grains (DGS). *T. reesei* can synthesize cellulase and has the ability to hydrolyze distiller’s grains and provide reducing sugar from cellulose during fermentation in mixed culture. The establishment of a one-pot solid-state fermentation for erythritol production involved the co-cultivation of *Y. lipolytica M53-S* with *T. reesei* Rut C-30, with the latter being inoculated with a 12-h delay. This approach resulted in both efficient saccharification of DGS and enhanced erythritol production simultaneously. Inoculation ratio 10:1, showed maximum production of erythritol up to 267.1 mg/gds from 200 g DGS in one pot SSF under optimized conditions (Liu et al., 2022).

Fatty acid ethyl esters (FAEEs), also known as biodiesel, can be a potential alternative for industrial fuel. FAEE can be synthesized through the trans-esterification of fatty acids with ethanol (Dunn et al., 2015; Santana et al., 2016). Previous studies have proven that *Y. lipolytica* can synthesize and accumulate large amounts of lipids (Back et al., 2016; Katre et al., 2017). A synthetic microbial co-culture system involving *Yarrowia* with *S. cerevisiae* was developed for FAEEs production. In this strategy, a genetically engineered *Y. lipolytica* strain was employed, which contains heterologous expressed wax synthase gene MhWS, while *S. cerevisiae* acted as ethanol supplier to produce FAEE biodiesel at a yield of 4.8 mg/L using optimized cultivation parameters (Yu et al., 2020).

Microbial bioremediation has been proven to be a very competent solution for environmental conservation by using different strategies to degrade pollutants (Kapoor et al., 2021; Ramzan et al., 2023; Saravanan et al., 2023; Some et al., 2021). The wastewater surrounding oil mills contains a significant amount of oil and grease, as well as elevated levels of COD (chemical oxygen demand), BOD (biological oxygen demand), and phenolic compounds (Kanmani et al., 2015). Previous studies reported *Y. lipolytica* and *Candida* sp. as potential microbes for the decontamination of wastewater (Gonçalves et al., 2009; Lanciotti et al., 2005). *Y. lipolytica* and *C. rugosa* as monoculture and co-culture systems and extracellular enzyme preparations were successfully employed for biodegradation of wastewater from a palm oil mill. The most substantial removal of triglyceride (98.5%) and COD (60.3%) occurred in undiluted POME treated with the co-culture over a 120-h period. It was considered highly cost-effective and efficient strategy due to its elimination of the requirement for pre-prepared dilution and specific nutrient requirements (Theerachat et al., 2017)

. Tellurium and selenium are commonly present elements in copper and sulphur-containing rocks, wastes of metal treating factories, metallurgical industries, petroleum refineries, and coal burning (Sinharoy and Lens, 2020; Spinks et al., 2016; Wadgaonkar et al., 2018). Wastewater from industries, cultivated land and mine tailing are more likely to be exposed by these elements, which lead to environmental hazards in water and land (Harada and Takahashi, 2008). Conventionally, bacteria have been favoured for the bioremediation of Te and Se, but Hosseini et al. (2023) for the first time, used two yeast strains (*Y. lipolytica* and *T. cutaneum*) and their co-culture for the co-reduction of Te and Se. The study reported that the co-culture of yeast strains has great capability for reducing the contaminated surfaces and synthesis of Te–Se NPs (nanoparticles). Inhibitory activities were revealed by Te and Se, and their removal was predicted by the first-order kinetics model. The study concluded that co-reduction of Te and Se by this method is economical, sustainable and ecological and biosynthesis of Te–Se NPs is a leading step in nanotechnology, medicine and other industries.

### 1.6 Engineering of *Y. Lipolytica* for lignocellulose-based xylose utilization

Lignocellulosic biomass (LB) seems to be one of the potential low-cost feedstocks for microbial fermentations as it can be hydrolyzed and transformed into monosaccharides (Brandenburg et al., 2018; Marđetko et al., 2018). Biofuels and various molecules production through lignocellulose fermentation have enormous potential for industrial applications owing to their economic practicality and environmental concerns. One of the challenges limiting the applications of lignocellulose is its high content of xylose, which is poorly metabolized by most microorganisms because carbon catabolite repression suppresses the assimilation of C5 sugars (Jin and Cate, 2017). Even though numerous microbes favor glucose as their primary carbon source, there are circumstances where xylose surpasses it, especially when specific metabolic demands must be addressed. Utilizing xylose as a carbon substrate enables the efficient production of new products that may be difficult to produce from glucose. Moreover, compared to glucose, xylose has a substantially higher metabolic flux through the pentose phosphate pathway (PPP), TCA cycle, and acetyl-CoA biosynthesis (Kwak et al., 2019).

**Figure 3:**
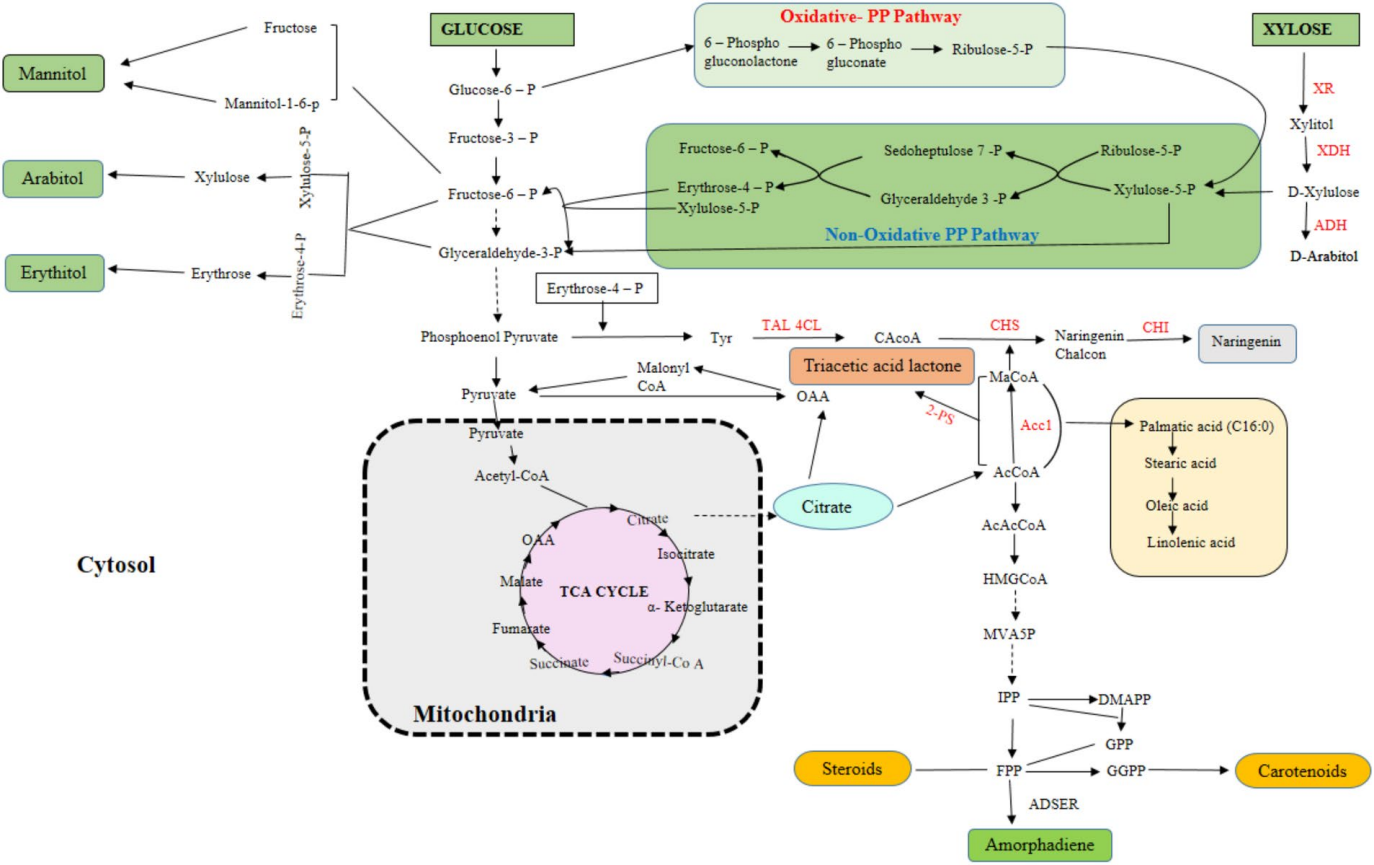
Overview of glucose and xylose assimilation pathways in *Y. lipolytica* for synthesis of various metabolites.

*Y. lipolytica* is a viable industrial chassis for transforming lignocellulose biomass into a variety of valuable products. Over the past three decades, the development of genetic engineering techniques has considerably benefited from the availability of completely annotated and sequenced genomes of *Y. lipolytica* strains and efficient transformation protocols (Liu and Alper, 2014; Shabbir Hussain et al., 2016). Additionally, several researchers have studied it from the perspective of systems biology using various omics data (metabolomics, proteomics, transcriptomics, and fluxomics) (Pomraning et al., 2015), which collectively allows metabolic engineering of *Y. lipolytica.* A very recent review provides a detailed description of all the genetic tools and strategies suitable for *Y. lipolytica* engineering (Ma et al., 2020). Since *Y. lipolytica* can accumulate acetyl-CoA and malonyl-CoA, it is typically the preferred host for the synthesis of fatty acids, terpenoids, flavonoids, and other compounds that need acetyl/malonyl-CoA as a precursor (Figure 3; Abdel-Mawgoud et al., 2018). The potential of this host has been extensively studied in recent years for the biosynthesis of valuable compounds from various carbon sources. However, despite efforts to better understand and improve xylose metabolism in this yeast, little research has been done on the biosynthesis of natural products based on xylose (Wei et al., 2020). Several studies have tried to broaden the substrate range of *Y. lipolytica* to utilize sugars derived from biomass, but it is not an effective catabolizer of xylose. However, the advancement of metabolic and genetic engineering techniques made it possible to use this yeast as a platform organism to convert xylose, the second most abundant substrate, into a variety of useful chemicals (Figure 1; Sun et al., 2021).

The xylose catabolizing pathway, in addition to important transporters, depends on the activity of three enzymes: (1) Xylose reductase (XR), which facilitates the conversion of xylose to xylitol; (2) xylulose dehydrogenase (XDH), which is involved in conversion of xylitol to xylulose; (3) and xylulose kinase (XK), which catalyzes the final conversion to xylulose-5-phospate. Microorganisms capable of catabolizing xylose commonly use two distinct pathways to transport xylose into the cytoplasm and transform it into xylulose. (1) Xylose reductase and xylulose dehydrogenase (the XR-XDH pathway), and (2) Xylose isomerase (XI) pathway (the XI pathway) (Figure 3). Researchers incorporated all xylose-degrading pathways in *Y. lipolytica* for xylose utilization. However, the modified strain with XI expressing pathway typically showed lower cell growth and slower rates of xylose assimilation in comparison to strains that express the XR-XDH pathway (Li and Alper, 2016). Due to its high metabolic flux and ease of expression, the XR-XDH route is the most studied in non-xylose-fermenting recombinant yeasts such as *Y. lipolytica* and *S. cerevisiae* (Wu et al., 2019). Although all xylose-degrading enzymes have been reported in *Y. lipolytica* system, however unable to grow sufficiently on xylose, thus indicating the need of genetic engineering (Rodriguez et al., 2016a; Ryu and Trinh, 2018). Apart from introducing homologous and heterologous xylose utilization pathways, other metabolic engineering techniques (Wu et al., 2019); lab adaptive evolution (Zhou et al., 2020); strain mating (Li and Alper, 2020), and artificial chromosome technology have been applied recently to improve xylose utilization in *Y. lipolytica*. Additionally, CRISPR tools (Schwartz et al., 2018) or artificial chromosomes (Guo et al., 2020) have also been employed to improve the ability of this yeast to degrade and utilize LB-based compounds as carbon source. Following are the few genome editing and metabolic engineering techniques that are commonly employed to facilitate the growth of *Y. lipolytica* on xylose derived from LB.

Recent progress in xylose metabolism engineering has significantly expanded the capacities of *Y. lipolytica* beyond what was described in earlier studies such as Bellou et al. (2016), Park et al. (2020), and Liu et al. (2022). While foundational work focused on introducing heterologous XR–XDH pathways, optimizing transporter systems, and addressing cofactor imbalances, recent researches (Choi et al., 2023; Das et al., 2024; Maurya et al., 2023; Xu et al., 2025; Zhang et al., 2026) has revealed the presence of previously underappreciated native xylose assimilation elements. Several updated studies have identified cryptic pentose-phosphate-associated enzymes, low-level endogenous xylitol dehydrogenase activity, and regulatory modules that may contribute to weak but detectable xylose flux, thereby modifying earlier assumptions that *Y. lipolytica* completely lacks native xylose metabolism (Jiang et al., 2024; Li et al., 2024; Yu et al., 2025).

Alongside these insights, major advancements in genome editing technologies have reshaped engineering strategies. The introduction of next-generation CRISPR systems, including Cas12a-based multiplex editing, high-precision base editors, prime-editing tools, and genome-wide gRNA libraries, has enabled more controlled and systematic modification of xylose-related genes such as xylA, xylB, tkt, tal, zwf, and other PPP-associated loci (Yu et al., 2025; Zhang et al., 2026). These tools complement and enhance the earlier genetic engineering approaches, enabling rapid construction of cofactor-balanced XR variants, optimized XDH systems, and transporter-overexpression strains with significantly improved xylose uptake rates (Das et al., 2024; Elsharawy and Refat, 2024; Jiang et al., 2024).

Recent studies have also emphasized the importance of transport engineering, introducing structure-guided redesign of xylose transporters and hybrid transport systems that surpass the performance of earlier heterologous transporters described in previous reports (Das et al., 2024; Li et al., 2024; Yu et al., 2025) Coupling these advances with systems biology–driven strategies, including genome-scale metabolic modeling, synthetic promoter engineering, and CRISPR-assisted adaptive laboratory evolution, has produced strains with markedly improved carbon flux distribution across the pentose phosphate pathway (Yu et al., 2025). These multicentric engineering approaches not only strengthen the earlier findings already discussed in the manuscript but also provide a contemporary and comprehensive understanding of xylose assimilation in *Y. lipolytica*, highlighting its growing potential as a chassis for lignocellulosic bioconversion (Xu et al., 2025; Zhang et al., 2026).

### 1.7 Co-utilization of glucose and xylose

*Y. lipolytica* can grow on a variety of hydrophilic and hydrophobic carbon sources, including fatty acids, alkanes, glucose, fructose, mannose, glycerol, and others (Mirończuk et al., 2016). Recently, various efforts have reported the efficient growth of *Y. lipolytica* on xylose by expressing a combination of genes such as XR, XDH, and XK (Ledesma-Amaro et al., 2016). This yeast has also exhibited a synergistic effect of mixed-sugar utilization (Ryu and Trinh, 2021), wherein xylose could be utilized in the presence of a glucose-xylose mixed sugar feedstock. This finding suggested that the presence of other sugars, such as glucose, may activate the expression of genes involved in the catabolism of xylose (Ong et al., 2019).

Native pentose-specific transporters have been identified in *Y. lipolytica* to activate the dormant pentose metabolism. Among 16 putative pentose-specific transporters, two specific transporters (YALI0C04730 and YALI0B00396) involved in xylose metabolism were overexpressed along with the rate-limiting d-xylitol and l-arabitol dehydrogenases. The xylose assimilation by the resultant strain was improved by about 23 to 50% in comparison to the parental engineered strain having overexpressed endogenous XD gene only. It was concluded that xylose and arabinose can be co-metabolized by this yeast, as arabinose share transporters and metabolic enzymes involved in some certain intermediate steps in the xylose assimilation pathway (Ryu and Trinh, 2018). Studies on the co-utilization of glucose and xylose indicated that xylose may be transported across the yeast cell membrane by glucose transporters in an unspecific manner (Sedlak and Ho, 2004).

Moreover, the upregulation of 13 additional transporters indicated the possibility of multiple transporters assisting in the transport of D-xylose across the cell membrane in *Y. lipolytica* for assimilation (Nicaud et al., 2017). It has been observed that the glucose presence in dual substrate cultures can inhibit the transport of xylose, so to solve this problem, Li et al. engineered ciGXS1 transporter through directed evolution to enable the simultaneous metabolism of glucose and xylose. This resulted in a transporter that can co-utilize glucose and xylose without inhibition. This strategy might be helpful in conditions where the transport of xylose is a limiting factor, such as when a high concentration of xylose is present or when different substrates are being used (Spagnuolo et al., 2018). All these attempts to engineer a native or hybrid xylose metabolic pathway in *Y. lipolytica* resulted in a recombinant strain with best ability to grow on LB. However few studies reported carbon catabolite repression (CCR) in engineered stain when grown in mixed sugars medium, where D-glucose severely inhibited xylose consumption. The majority of microorganisms (Görke and Stülke, 2008) exhibit this phenomenon, which may be related to inefficient xylose transporters, intricate metabolic regulation, and the dormant pentose phosphate pathway (Ryu and Trinh, 2018). However, metabolic and evolutionary engineering can reduce or even completely remove CCR (Bruder et al., 2015). For example, mutants with repressed pentose metabolism were constructed through four stages of adaptive laboratory evolution ALE of *Y. lipolytica* in a medium containing xylose and the D-glucose analogue, 2-deoxyglucose (dG), for 64 days. Since dG cannot be metabolized to produce energy, microbes have to adapt in order to use less energy for catalytical conversion of dG and D-glucose. This has been achieved by downregulating the activity of hexokinase and the hexose metabolism pathway. Based on these findings, it was concluded that utilization of non-metabolizable substates and blocking the hexose metabolic pathway produced the conditions that led microorganisms to evolve to transport and use xylose in the presence of D-glucose (Zhou et al., 2020).

Recent developments in microbial lipid biorefineries have expanded the landscape of oleaginous microorganisms beyond traditional yeasts and moved toward integrated, process-level strategies for sustainable biofuel and biochemical production from lignocellulosic biomass. Al Azad et al. (2024) comprehensively review these new trends, highlighting innovations in pretreatment methods, enzymatic hydrolysis of biomass, fermentation models (e.g., separated hydrolysis and lipid production, simultaneous saccharification and lipid production, solid-state fermentation), and metabolic-engineering techniques across a variety of oleaginous microbes (Al Azad et al., 2024). Within this broader context, *Y. lipolytica* stands out as a particularly attractive chassis thanks to its metabolic plasticity, lipid-accumulation capacity, and extensive synthetic biology toolbox. Recent work using lignocellulosic hydrolysates (e.g., rye straw) has demonstrated that engineered *Y. lipolytica* strains can efficiently convert xylose-rich waste streams into lipids, confirming their suitability for second-generation biorefineries.

### 1.8 CRISPR-based genome editing

CRISPR/Cas systems can be classified into three groups based on versatile Cas proteins: knock-out/in-oriented CRISPR/Cas9, CRISPR activation (CRISPRa) and CRISPR interference (CRISPRi) (Sharma et al., 2017). This genome editing technique has been effectively employed by various researchers to produce more valuable compounds in *Y. lipolytica*, making it a potential candidate for metabolic engineering research (Abdel-Mawgoud and Stephanopoulos, 2020). For example, CRISPR-based activation system (CRISPRa) was employed to upregulate genes involved in cryptic cellobiose degradation that facilitate *Y. lipolytica* growth on the biomass-derived sugars. This eliminates the need for cloning overexpression cassettes or expressing heterologous enzymes (Ryu et al., 2016). Firstly, the effectiveness of various transcriptional activators was compared in *Y. lipolytica,* among which synthetic tripartite activator VPR produced the highest activation, which was subsequently fused with dCas9, which was directed to various locations in a synthetic promoter, driving hrGFP expression to achieve activation. Then, two distinct native *β*-glucosidase genes were transcribed using the CRISPRa system, enabling improved yeast growth on cellobiose as sole carbon source (Schwartz et al., 2018). Thus, it is possible to investigate the encoded functions of additional silent regions in the *Y. lipolytica* genome by turning them on.

CRISPR/Cas9 has also been used to destroy XK and XD by introducing frameshift mutation to demonstrate that both genes are important for xylose/xylitol assimilation in *Y. lipolytica* (Rodriguez et al., 2016a; Xia et al., 2023). Based on these findings and additional research conducted on *E. coli,* scientists engineered xylose utilization pathway of this yeast, allowing it to grow robustly on xylose (Shi et al., 2018). CRISPR/Cas9-based modification of xylose metabolism in *Y. lipolytica* was achieved by integration of ss*XR*, ss*XDH*, and yl*XK* gene expression cassettes in 3 different loci to improve the ability for limonene production. The best strain with HMG*1* and *ERG12* overexpression produced *a* significant amount of limonene when grown on fermentation media containing lignocellulose hydrolysate (Yao et al., 2020).

### 1.9 Promoter engineering

In the field of synthetic biology, promoters stand out as crucial components, and for eukaryotes, effectively regulated promoters play a vital role in controlling gene expression. In order to better understand their regulatory mechanism and their impact on transcription, numerous researchers exploited *Y. lipolytica* genome to identify and characterize endogenous promoters. This effort also significantly aided in the advancement of promoter engineering. Since precise gene expression is necessary to ensure optimal metabolic flux in pathway engineering and prevent an excessive metabolic burden, promoters with variable transcriptional abilities or required properties are an indispensable tool for synthetic biology (Sun et al., 2022). Common promoter elements in yeast typically consist of the TATA box, core promoter, and upstream activation sequences (UAS). The activity of promoters can be adjusted logically by modifying these elements. Blazeck et al. (2013) reported that the combination of different UAS elements (UASTEF and UAS1B) in *Y. lipolytica* resulted in seven-fold higher expression levels of synthetic promoters as compared to unmodified promoter. It was reported in a recent study that by overexpressing endogenous or heterologous *XR*, *XDH*, and combining with endogenous *XK* under the control of TEF promoter, *Y. lipolytica* strains could produce 16.5 g/L of lipids from xylose-rich agave bagasse hydrolysate (Niehus et al., 2018). Following this, hybrid synthetic promoters (TEFin) was constructed which triggered the transcriptional activation of genes of interest in *Y. lipolytica* after the addition of xylose. In order to increase the lipid accumulation with effective growth on a xylose substrate Drzymała-Kapinos et al. (2022) carried out the minimal genetic modification of *Y. lipolytica* by over expressing endogenous *XDH* and *XK* for stable yeast growth, reaching almost 5.5 g/L. While overexpression of *DGA1* enhanced lipid production up to 2.19 g/L which was 10 folds higher in comparison to wild type strain by utilizing xylose-rich rye straw hydrolysate. All genes were driven by synthetic hybrid promoter UAS1B16-TEF.

### 1.10 Strain mating

Strain mating is a conventional genetic method that has found widespread application in both fundamental genetic research and industrial breeding approaches. In *Yarrowia*, only one mating locus is reported, and there are two mating types exist: type A and type B, which are determined by the codes in this unique locus (Barth and Gaillardin, 1997). The strain mating approach was also employed to engineer the xylose assimilation pathway *in Y. lipolytica* to produce riboflavin and 𝜶-linolenic acid (ALA) (Cordova and Alper, 2018; Wagner et al., 2018) both of which have strong antitherapeutic effects on human health. Li and Alper (2020) have discovered that strain mating can yield xylose-utilizing strains that can produce valuable biomolecules from xylose at a higher rate than the parental strain. Strategically they mated engineered and adapted xylose-utilizing strain of *Y. lipolytica* with riboflavin, ALA, and Triacetic acid lactone TAL-producing engineered strain to obtain diploid strains that can quickly and directly produce these molecules from xylose. For this they employed a piggyBac transposon system to convert the strain to type B and overexpressed the endogenous XK gene, which is driven by a strong constitutive promoter in a high xylose-utilizing lipid-producing strain in order to further increase catabolic rates. Following that, they crossed the modified strain type B with various strains of type A that produce riboflavin, ALA, and TAL. More specifically it was also observed that overexpressing the native *ylXKS* increased cell growth on glucose as well as xylose. However, they concluded that specific productivities were decreased due to improvement in cell growth by the obtained diploid strains.

### 1.11 Production of valuable metabolites

Remodeling the metabolism of *Y. lipolytica* and other supporting microorganisms to utilize alternative substrates is anticipated to enhance the metabolization of waste or by-products that are readily processed by wild-type strains (Madzak, 2021). Furthermore, this metabolic adaptation is expected to extend the potential for valorization to a significantly broader range of feedstocks derived from diverse industries. Genetic engineering can permit the use of lignocellulosic hydrolysates from various industries in addition to the use of waste cooking oils and crude glycerol, both of which are reported as natural substrates for *Y. lipolytica*. In this section few industrially important metabolites produced by genetic engineering of this yeast using xylose or LB are discussed.

**Terpenoids** are considered a very vital group of compounds necessary for human health. They have a number of significant applications not only in human nutrition but also in animal health, cosmetics, improving agricultural yields, biofuels, flavouring, and disease treatment. Moreover, they are also involved in various metabolic processes (Bon et al., 2018; Li C. et al., 2023). *Y. lipolytica* can be genetically modified to produce a variety of terpenoids through incorporating terpene synthase from different heterologous sources, by increasing cytosolic acetyl-CoA supply, overexpressing MVA pathway genes, modulating the cofactor supply and hydrophobicity to accumulate terpenoids and fine tuning the expression of key enzymes involved in terpenoid production. **Limonene**, a valuable monoterpene, found in more than 300 different plant varieties exhibiting anti-microbial properties and therapeutic attributes which makes it a chassis compound for pharmaceutical industry. It was produced in an engineered *Y. lipolytica* strain by heterologous overexpression of limonene biosynthesis genes in combination with overexpression of XR and XDH from *Scheffersomyces stipites* and native XK under hp4d promoter. The resulting strain produced a limonene titer of 9.00 mg/L using xylose as substrate after overexpressing endogenous HMG-CoA reductase (HMG1) and mevalonate kinase (ERG12) genes in the MVA pathway and increasing the gene copy numbers of the limonene synthetic pathway. Additionally, using 50% detoxified lignocellulosic hydrolysate, the maximum limonene titer reached 20.57 mg/L (Yao et al., 2020). **Protopanaxadiol** (PPD) is active triterpenoid naturally present in *Panax ginseng*. This compound exhibits promising activity and minimal toxicity to normal cells, making it a promising candidate for both antidepressant and antineoplastic treatment (Zhao et al., 2017). This chemical compound was produced in *Y. lipolytica* from xylose as substrate by first introducing codon-optimized XR-XDH pathway-related genes from *S. stipites* along with native XK gene into the genome. Furthermore, evolutionary adaption strategy was employed to enable the modified strain to grow on xylose effectively. After that, a PPD synthesis fusion expression module was constructed, and integrated into the genome of *Y. lipolytica* resulting in 60.1 mg/L of PPD using xylose as substrate. They further overexpressed the genes of the MVA pathway and the endogenous xylose transporter (TX), transaldolase (TAL) and transketolase (TKL) to support xylose metabolism to boost the PPD titer to 88.73 mg/L. The PPD titer was increased to 300.63 mg/L with a yield of 2.505 mg/g xylose and a productivity of 2.505 mg/L/h in a 5-L bioreactor after the fermentation conditions were optimized (Wu et al., 2019).

**Naringenin** is a bioactive polyphenols and precursor for many flavonoids with various pharmacological and biological effects on human health. In an attempt to produce this compound in *Y. lipolytica,* researcher developed an inducible activator (VPRHX) to dynamically control the expression of *Xr, Xdh, Xks, and Xt*. Using xylose as the substrate, the final strain generated roughly 715.3 mg/L of naringenin in shake-flask cultures linking the biosynthesis of naringenin and xylose utilization (Wei et al., 2020).

***β*-ionone** is another valuable compound and has been produced in *Y. lipolytica* strain through genetically engineering by overexpressing *PhCCD1* genes coding for carotenoid cleavage dioxygenase enzyme, which is involved in β-carotene cleavage. The resulting strain was able to successfully grow on hydrolysate of food and sugarcane bagasse, producing 4 g/L and 1.93 g/L of β-ionone, respectively, (Chen et al., 2022). Similarly, β-farnesene production was improved in this yeast by upregulation of MVA pathways genes along with β-farnesene synthase. The modified strain was able to produce 7.38 g/L of β-farnesene when grow on lignocellulosic hydrolysate (Bi et al., 2022).

Three different valuable biomolecules such **as riboflavin, ALA, and TAL** were produced from xylose in *Yarrowia* by strain mating approaches as mentioned above. Surprisingly, obtained diploid strains were able to maintain production levels by xylose that were either comparable to or greater than those of glucose. This diploid strain produced 96.7 mg/L of riboflavin, 0.52 g/L of ALA, and 2.9 g/L of TAL at the flask scale cultivation using xylose as substrate (Li and Alper, 2020). More recently better production of TAL was achieved in *Y. lipolytica* by investigating multiple metabolic engineering strategies for efficient conversion of xylose. Firstly, xylose assimilation was modulated, resulted in more NADPH generation. pH control improved the supply of malonyl-CoA, by fine tune expression of acetyl-CoA carboxylase gene. Finally, FAS pathway was blocked by using cerulenin, resulting in 5.03 g/L of TAL when pure xylose was used a substrate while growth on wheat straw hydrolysate produced 4.18 g/L of TAL (Liu et al., 2023).

**Lipids** Since *Y. lipolytica* is an oleaginous yeast, it has the ability to produce lipids by using various carbon sources. For instance, xylose assimilation pathway was improved in *Y. lipolytica* by co-expression of heterologous XR and XD and starvation adaptation and resulting strain produced over 15 g/L of lipid in bioreactor. The genomic sequencing and genetic analysis revealed that genomic duplications occurred with increased expression level due to starvation (Li and Alper, 2016). Later on, Do Yook et al. (2020) co-expressed mutant XI (*XylA3*) and native XK gene to avoid cofactor imbalance. Xylose utilization was then strengthened by deleting peroxisome biogenesis factor 10 (*PEX10*) to prevent lipid degradation and by overexpressing the diacylglycerol acyltransferase gene (*DGA1*) to direct metabolic flux towards lipid production and obtained highest yield, 12.01 g/L of lipid from lignocellulosic hydrolysates. Similarly, *Y. lipolytica* was engineered to metabolize xylose for the production of citric acids and lipids. It was shown that overexpressing XR and XDH genes from *S. stipites* were essential for xylose catabolism but the cell growth was not ideal but combining with overexpression of endogenous *XK* resulted in improved ability of engineered strain to show same growth profile as of wild type strain in glucose. The modified strain converted xylose into 80 g/L of citric acid and 20–50 g/L of lipid production when xylose was co-fed with glycerol in a 5 L bioreactor (Ledesma-Amaro et al., 2016). Similarly, Niehus et al. (2018) created a strain of that could use xylose through overexpression of endogenous XDH, XR and XK genes. Further series of modification were done in xylose utilizing strain for lipid production by combining the deletion of the endogenous fatty-acyl-CoA oxidase POX1-6 and triacylglycerol lipase 4 TGL4 coding genes with the overexpression of the endogenous DGA2 and G3P dehydrogenase GPD1 coding genes. To further increase the supply of acetyl-CoA phosphoketolase pathway enzyme were overexpressed. When final engineered strains were grown on lignocellulosic hydrolysate, 16.5 g/L of lipids was produced with a yield of 0.344 g/g and a productivity of 0.185 g/L/h.

Most recent proteomic study on *Y. lipolytica* conventional CBS7504 and undomesticated YB420 strain demonstrated the differential ability of both strains to grow on switchgrass hydrolysate. It was observed that YB420 produced high levels of lipids 1.6 g/L by using xylose in hydrolysate without affecting the growth while degradation of cell biomass and lipid was reported in CBS7504 when xylose remained as sole carbon source. Proteomic analysis revealed the upregulation of various PPP proteins in YB420 such as *Xyl2* and *Xyl3,* essential for xylose transport through the PPP. While lipid degradation, decreased cell mass, and increased xylitol secretion were reported in strain CBS7504 with the nearly unchanged protein abundances in PPP (Walker et al., 2021).

In most recent study a “Push−Pull−Block” strategy was employed to increase the lipid production capacity of *Y. lipolytica*. Firstly, push strategy was conducted by overexpression of *ACC1* which increased malonyl-CoA supply, followed by pull strategy in which additional copy of *DGA1* was integrated to redirect the acyl -CoA flux towards TAGs synthesis. Subsequently, the Block strategy involved the knockout of *TGL4* and *PEX10* to stop the degradation of TAGs and acyl-CoA. After that, heterologous *XR, XDH* and native *XK* genes coexpression was used to establish the xylose utilization capacity followed by Adaptive laboratory evolution ALE. This resulted in 16.25 g/L of lipid production from xylose substrates (Sun et al., 2023).

**Succinic acid (SA)** is another valuable chemical with various industrial applications. Its global demand was 50,000 metric ton in 2016 and predicted to get double by 2025 (Babaei et al., 2019). Recent study conducted metabolic evolution of *Y. lipolytica* PGC01003 to generate evolved strain PSA02004, resulting in the restoration of glucose metabolism and an increase in the rate of glucose uptake for SA fermentation. This strain PSA02004 was then investigated for mixed substrate utilization of glucose and xylose for SA production with reported productivity of 28.2 ± 0.6 g/L. Further cultivation of this strain produced 33.2 ± 0.3 g/L of SA using sugarcane bagasse hydrolysate as carbon source (Ong et al., 2019). In extension to this study, *Y. lipolytica* PSA02004 strain was also genetically engineered for SA production by overexpressing a pentose pathway cassette that contains the genes for XR, XDH, and, XK under TEF promoter. The resulting strain produced a significant amount of SA (11.2 g/L) and showed strong growth 7.3 g/L when given xylose as only carbon source in batch fermenter. Comparable results were obtained when recombinant strains were grown on sugarcane bagasse. Fed batch fermentation reported a progressive drop in pH below 4.0, with biomass concentration of 11.8 g/L and 22.3 g/L of SA. The enzyme activity analysis showed that slightly high activity of XDH as compared to XR facilitated better synchronization between two enzymes, resulting in efficient conversion of xylose to xylulose with no xylitol accumulation as by-product (Prabhu et al., 2020).

### 1.12 Conclusion and future prospects

By examining the diverse applications of *Y. lipolytica* in bioprocessing and bioconversion, this review highlights its potential as a robust and flexible microbial platform for the sustainable production of high-value compounds. The strategic use of artificial microbial consortia and co-culture systems has improved its metabolic output, enabling the synthesis of volatile aromatics, citric and succinic acids, lipids, and other industrially relevant molecules. These advancements underscore the critical role of interspecies interactions and demonstrate how *Y. lipolytica* can effectively convert varied feedstocks into value-added products. Genetic engineering approaches, particularly CRISPR/Cas9, continue to accelerate progress by enabling targeted enhancement of metabolic pathways. The incorporation of xylose utilization modules, combined with adaptive laboratory evolution, has markedly improved lipid production from xylose, broadening the organism’s suitability for lignocellulosic bioprocessing. Expanding CRISPR-based tools for both conventional and non-conventional yeasts has opened new directions for metabolic rewiring, while promoter engineering remains essential for achieving finely tuned gene expression and pathway control.

The application of “Push–Pull–Block” metabolic engineering demonstrates how coordinated overexpression, flux redirection, and gene deletion can substantially elevate product yields in *Y. lipolytica*. The integration of enhanced xylose pathways and adaptive evolution further strengthens its performance in biomass-derived carbon streams. Its capacity for bioremediation, including pollutant degradation and participation in integrated fungal–bacterial systems for lignocellulose conversion, further underscores its versatility for circular bioeconomy applications. Looking ahead, the development of advanced co-culture systems involving *Y. lipolytica* and complementary microorganisms holds considerable promise. Deeper investigation of microbial synergy could expand metabolic diversity and support the biosynthesis of an even broader portfolio of high-value products. Identifying novel consortia and understanding interaction dynamics will be crucial for designing more efficient and resilient bioprocesses. At the same time, future research must address practical barriers such as scale-up complexity, economic feasibility of lignocellulosic feedstocks, and contamination risks especially in minimally sterilized or open co-culture environments. Continued refinement of the genetic toolkit, enabling more precise pathway manipulation, will support the creation of tailored strains with enhanced industrial productivity and pave the way for broader deployment of *Y. lipolytica* in large-scale sustainable biomanufacturing.
